# Examining the Features of Parks That Children Visit During Three Stages of Childhood

**DOI:** 10.3390/ijerph16091658

**Published:** 2019-05-13

**Authors:** Elliott P. Flowers, Anna Timperio, Kylie D. Hesketh, Jenny Veitch

**Affiliations:** Institute for Physical Activity and Nutrition (IPAN), School of Exercise and Nutrition Sciences, Deakin University, Geelong 3220, Australia; anna.timperio@deakin.edu.au (A.T.); kylie.hesketh@deakin.edu.au (K.D.H.); jenny.veitch@deakin.edu.au (J.V.)

**Keywords:** parks, playgrounds, children, local green space, park features

## Abstract

Parks provide an opportunity for children to be physically active, but are rarely fully utilised. A better understanding of which park features attract children of varying ages is needed. This study examined which features are present at parks that children visit most often at different stages throughout childhood. Parents reported the park their child visited most often at three timepoints: T1 = 3–5 years, T2 = 6–8 years, and T3 = 9–11 years. These parks were then audited (using a purposely created audit tool) to capture information relating to access, activity areas and quality. Online mapping tools were also used to determine walking distance to parks and park size. Parks visited at T2 were further from home, larger, and had more road crossings, full courts, other facilities and comfort amenities such as toilets and lights than T1 parks. Parks visited at T3 were larger and had more sports ovals compared to T1 parks, and were significantly less likely to have barbeque facilities than T2 parks. Our findings suggest that as children transition from pre-school (T1), to primary school age (T2 and T3), they visit parks that have more facilities to support sport and active recreation.

## 1. Introduction

Australian children are generally not sufficiently active; with only 34% of 2–5 year olds, and 17%–40% of primary school age children meeting the recommended physical activity levels [1]. Childhood activity levels in Australia are roughly equal to some high income westernised countries (Finland, Germany, United States of America) [2], but below that of others (Canada, England, the Netherlands etc.). The Australian government recommends that preschool children (aged 3–5 years) should spend at least 180 min a day in a variety of physical activities, of which 60 min is energetic play such as running, jumping, kicking and throwing, spread throughout the day. Older children (aged 5–12 years), should accumulate at least 60 min of aerobic activities, of which some should be of vigorous intensity [3]. Increasing childhood physical activity levels is a public health priority in Australia and many other developed countries.

The World Health Organization recognises the importance of green spaces in urban areas for physical activity: ‘Urban parks and gardens provide routes for walking and cycling for transport purposes as well as sites for physical activity, social interaction and for recreation’ [4]. In developed countries, parks afford children the opportunity to be physically active: grassy areas provide space to run and throw, courts and ovals mark the boundary for sports (Australian rules football, cricket, soccer etc.), and playground apparatus facilitate climbing, sliding and swinging [5]. Analysis of children’s park use shows that parks are used for sedentary, light, and moderate- to vigorous-intensity physical activities (MVPA) during evenings on weekdays and on weekends [6], indicating that children’s park use is varied. Furthermore, being active in nature has been shown to elicit physiological and psychological benefits over and above being active in urban areas [7], and children are more active when given access to natural environments for play [8]. Despite this, parks are generally not a fully utilised location for physical activity thus there is significant potential to increase active use of parks [9,10].

Research suggests that certain amenities within parks are associated with overall visitation and physical activity among children and adolescents [11,12,13]. Park characteristics include physical features such as facilities and amenities (i.e., paths, lighting, play equipment, toilets, water fountains), size, access, aesthetics (i.e., landscaping, maintenance), incivilities (i.e., rubbish and graffiti), and social factors such as the presence of others and personal safety. When examining public open spaces and children’s physical activity levels, Timperio et al. [12] found that the presence of playgrounds at the closest public open space to home was positively associated with boys’ and girls’ (8–9 years old) MVPA outside of school hours. Similarly, Besenyi, et al. [14] found that young people with a playground within a half-mile (800 m) from home were 2.5 times more likely to meet physical activity recommendations, compared to those without a nearby park. Qualitative studies have also suggested that the perceived presence of play equipment and facilities at parks are important factors for encouraging both children and parents to be active [15,16,17,18]. In support of this, an observational study showed that 12 months after the installation of a new play-scape, park visitations by children increased by five-fold and children were over four times more likely to engage in MVPA, compared to a control park that did not receive a new play-scape [19].

Distance from home to the nearest park and park size may also influence park visitation. A study in Southern California, USA, revealed that for children (8–14 years) the odds of extended park use (>15 min) increased four-fold when the distance between home and the nearest park decreased by 100 m [20].We also know that children do not necessarily visit the closest park to home [21] and it is possible that the features within the park attract them to visit parks located further away. In support of this, a recent review found that park attractiveness was positively associated with children’s (3–12 years) independent mobility [22]. To our knowledge, no studies have examined if park size or distance to parks and park visitation varies as children get older.

Much of the research relating to parks and physical activity has focused on adults or a broad age-range within youth populations. However, some research has highlighted differences in the relationship between park features and physical activity levels for younger children compared to older children For example, one study found that the number of playgrounds in the local area was positively associated with younger boys’ physical activity, but there was only a non-significant trend (*p* = 0.07) among adolescent boys [23]. Similarly, a recent review found that associations between physical activity and objectively-measured recreational facilities (access, density, proximity to recreation/sports facilities) were more consistent for younger children (3–12 years old), than adolescents (13–18 years old), however, associations between access to parks and physical activity were inconsistent for both age groups [24]. This suggests that for children, features of parks (including playgrounds) are an important factor for visitation; however, to our knowledge no studies have examined if the features present in parks that children visit vary as children age. 

Improving parks and providing amenities that encourage children to visit parks and engage in park-based physical activity is potentially a long-term and sustainable way to increase levels of physical activity. It is therefore important to obtain a greater understanding of which park features may attract children at different stages of childhood to visit parks. Therefore, the aim of this study was to investigate the features present in parks that children visit at different ages. It was hypothesised that as children get older, they are more likely to visit parks with less playground apparatus or more challenging playground equipment, and more features for organised sports (such as courts and ovals), to reflect changes in their physical activity and play behaviours. In this regard we also expected older children to visit parks that were larger (to accommodate more open space and sports facilities), and further away.

## 2. Materials and Methods 

Data for this study were collected as part of the Healthy Active Preschool and Primary Years (HAPPY) Study, which has been described previously [25]. In short, parents of children 3–5 years of age were recruited from 64 preschools and 77 long day care centres across six local government areas (including low-, mid-, and high socioeconomic status) in metropolitan Melbourne. Staggered two-stage recruitment occurred at each of the three timepoints: T1 (age 3–5 years) spanned August–December 2008 and July–November 2009; T2 (age 6–8 years) spanned August 2011–March 2013; and T3 (age 9–11 years) spanned July 2014–March 2016. At T1, 766 participants consented to longitudinal contact. At T2, 567 participated (74%), and at T3, 571 participated (77%). For the current analyses, data for specific timepoints were excluded if participants had moved house between time points. For example, if participants moved house between T2 and T3, T1 and T2 data were included and T3 data excluded. The final analytical sample consisted of 375 participants. However, because of the excluded data there were 353 participants included at T1, 375 at T2, and 310 at T3.

As part of the T3 HAPPY study, parents were asked to complete an online survey (or mailed paper survey on request). Parents were asked ‘What is the park you and your child visit most often? (please provide as much information as you can; e.g., address or nearest crossroad/street/landmark)’. Additionally, responders were asked to retrospectively report which park their child visited most often when they were 3–5 years old, and 6–8 years old (matched to T1 and T2 age ranges). For all three items, responders were also able to indicate if they did not visit parks. In addition, parents reported their home address, their highest level of maternal education, marital status, the number and age of other siblings and the gender of their child at T1.

Reported parks were located using Google Maps (https://maps.google.com), a publicly available web mapping service that offers satellite, aerial and street view imagery. Previous research has found that Google Maps can be used remotely to accurately assess public open spaces (including parks) [26]. Using the information provided by the parents, parks were located using the inbuilt search function and the default aerial imagery. If the park(s) was unidentifiable, the participant was excluded (*n* = 3).

Walking distances from home to parks were calculated using Google Maps. Individually, home addresses (as WGS84 X, Y coordinates, which were converted from home postcodes) were entered as route origins, and park names were entered as route destinations. Next, the walking option was selected and the destination marker was dragged to the closest park access point from the child’s home. This overcame the default setting in Google Maps which navigates to a fixed point within each park (often the centre of the park). If multiple routes were displayed as viable walking options, the shortest route was selected, however this may not always be the quickest route due to intersections and path gradients. Whilst calculating walking distances, and using a trial and error approach, the closest park to the child’s home was also identified.

Park sizes were obtained from the Victorian Planning Authority’s (VPA) Metropolitan Open Space Network Portal [27]. This portal provides information on open space throughout Melbourne and is based on work from the Victorian Environmental Assessment Council’s (VEAC) Metropolitan Melbourne Investigation (2011), and information from Melbourne’s 32 metropolitan councils. The map based tool allows users to select individual parks within Melbourne and obtain sizes to the nearest 10 m^2^. Parks that were made up of multiple polygons (such as when a river or road intersected the park) were reported as the sum of all polygons.

The features of the parks were assessed using a purposely created pen and paper audit tool, which was adapted from other audit tools [12,28,29] to meet the requirements of this study. For this study, 317 parks were audited between September 2014 and May 2017. The audit tool consisted of 35 items divided into three sections: *access and surrounding neighbourhood; activity areas within the park;* and *park quality and safety* (see Table 1). The *access and surrounding neighbourhood* section contained six items relating to how easy it is for visitors to get to the park. For example; ‘Are there footpaths on any roads bordering the park?’ and ‘Is there a public transport stop within sight of the park?’. All six items were scored yes or no. The *activity areas within the park* section contained 10 items relating to features of the park that are conducive to physical activity (e.g., playground apparatus, sports ovals, and walking trails). Examples include ‘Number of full courts available’ and ‘Are any of the playgrounds suitable for under 5 year olds?’. Items were scored as whole numbers and yes/no responses as appropriate. The *park quality and safety* section contained 19 items relating to additional amenities (e.g., toilets, water fountains, etc.), safety (e.g., lights, vandalism, etc.), dog regulations and aesthetic qualities (artistic features, evidence of landscaping, etc.). Examples include ‘Are there picnic tables?’ and ‘Is there a sign prohibiting dogs from the playground area?’ and all 11 items were scored yes or no.

Seventeen parks were audited twice on different occasions by the same auditor to assess intra-rater reliability, and 32 parks were audited by two different auditors to assess inter-rater reliability. Using Cohen’s Kappa for binary variables, and intraclass correlation for count variables, the mean intra-reliability score was 0.88, and the mean inter-reliability score was 0.82. Scores above 0.7 are considered good [30,31].

### Data Analysis

A series of mixed-model tests (with participants as fixed effects, and park characteristics as random effects) were run to assess differences between the three timepoints. Mixed-model logistic regressions were used for variables with binary outcomes (such as whether the park had lights, or drinking fountains etc.), mixed-model negative binomial regressions were used for variables with over-dispersed count outcomes (such as number of full courts and ovals in the park) and mixed-model poisson regressions were used for variables with normally dispersed count outcomes (such as number of playgrounds). For the variables with scale data (i.e., walking distance to parks (m), and park size (hectares)), the data were log-transformed to account for non-normal distribution, and coefficients were reported in relation to geometric means, which are roughly equivalent to medians. Only variables that successfully converged during data analyses were included in Table 2. Statistical analysis was performed using Stata 14 SE (Statacorp, College Station, TX, USA) and statistical significance was accepted at *p* < 0.05.

## 3. Results

Parental responses yielded a sample of 210 boys and 165 girls. The majority (69%) of the children’s mothers were educated to degree level, and the remaining were educated to Year 12 or equivalent (25%), Year 10 (5%) or did not disclose (1%). The majority (54%) of the children’s parents were married/de-facto, and the remaining were separated/divorced/widowed (9%) or did not disclose (37%). The majority (85%) of children had at least one sibling and the average age of siblings at T1 (child age 3–5 years) was 10 years. Descriptive characteristics of the main park visited at each timepoint are shown in Table 1. Between 34%–41% of parks that children typically visited across the timepoints were closest to home. Between each timepoint, more than 30% of the sample usually visited different parks with children being less likely to visit the same park at T1 and T2 (62%), than they were at T2 and T3 (68%).

### Associations Between Park Features at Three Timepoints

*Access and surrounding neighbourhood*: significant differences were found for walking distances, park size, and road crossings, but not for the remaining variables (Table 2). In support of the hypothesis, T2 parks were more likely to be further away (expβ = 1.16), and larger (expβ = 1.15) compared to T1. T3 parks were also larger than T1 parks (expβ = 1.15). Additionally, parks at T2 were nearly twice as likely to have road crossing signals bordering the park compared to T1 (OR = 1.89). There were no differences between T2 and T3 parks.

*Activity areas within the park*: T2 parks had significantly more full courts (e.g., basketball, netball, tennis etc, IRR = 1.42), sports ovals (IRR = 1.36), and other facilities (e.g., athletics track, fitness station, swimming pool, IRR = 1.10), compared to T1 parks, which supports the hypothesis. Additionally, T3 parks had significantly more sports ovals than T1 parks (IRR = 1.33). No significant differences were found between timepoints for the remaining variables relating to activity areas within the park. There were no differences between T2 and T3 parks.

*Park quality and safety*: T2 parks had significantly higher odds of having toilets (OR = 1.63), and lights (OR = 1.69) compared to T1 parks, and T3 parks had significantly lower odds of having barbeque facilities (OR = 0.54), compared with T2 parks. No significant differences were found for the remaining variables relating to park quality and safety.

## 4. Discussion

This study aimed to identify the park features present in the parks that children of different ages visit. We found that the greatest differences in park features occurred between the parks reported at T1 (3–5 years) and T2 (6–8 years). As children transition from T1 to T2, the parks they visit most often tended to be further away and larger (based upon geometric means). These parks were often more equipped, particularly with sports and recreation facilities such as courts, ovals and other facilities (such as road crossings, toilets and lighting). We did not find any significant differences between the provision of playgrounds and playground equipment (amount and age appropriateness). The findings highlight that subtle differences exist between the types of parks child are visiting at different ages throughout childhood, particularly when transitioning from pre-school to early primary school age.

In keeping with previous research [5,12,16,24], the provision of playground equipment was an important factor for park visitation in this study. Across all three timepoints, the average park had at least one playground and 12 pieces of playground equipment (see-saws, slides, swings etc.). Additionally, the majority of the parks contained playground equipment suitable for children under the age of 10. This suggests that parents of young children are opting to take their children to parks that have sufficient age appropriate playground equipment. This supports previous research whereby the installation of new and exciting play equipment has been shown to increase park visitations [19]. We did not find that younger children visited parks with the most playground equipment, however, we did not assess the types of activities children performed when at the park. It is feasible that whilst there was no difference in the provision of playground equipment between timepoints, younger children may have been more likely to use the equipment than older children. Additionally, it is possible that parents with multiple children visit parks that have facilities suitable for a range of child ages.

Consistent with other studies [12,24,32], our findings suggest that sports courts, ovals and facilities are important features in the relationship between age and park visitation. In this study, children aged 6–8 years visited parks that had more courts, ovals and other facilities (athletics tracks, swimming pools etc.) compared with children aged 3–5 years. In contrast, no significant differences were found between T2 and T3, although at T3 there were more sports ovals at parks visited than at T1. This suggests that the biggest change in park features observed occurred between T1 and T2 (i.e., around the same time that children start primary school), which is consistent with our findings that more children changed the park they visited most frequently between T1 and T2, than between T2 and T3. Between each timepoint, more than 30% of the sample usually visited different parks. Therefore, urban planners should ensure that parks within each neighbourhood provide features the meet age related needs, particularly when renovating existing parks, or proposing new parks in urban redevelopments. In Melbourne, Australia, it is compulsory for children to be in full-time education from the age of 6 onwards and exposure to ‘games and sports’ is one of the 12 focus areas for the Victorian Curriculum Health and Physical Education [33]. Therefore, it is possible that as children start school, they also transition from play-based physical activities towards more formal sport based physical activities that require specific park features (such as courts and ovals). Parks that include ovals and courts are likely to be larger than parks without, and as a result are likely to be more sparsely distributed, particularly in dense urban areas. Our findings in relation to park size and walking distance support this. Parks at T2 and T3 were larger than those at T1, however the difference between 3.9 and 4.5 hectares to children is most likely negligible. Similarly, whilst the walking distance to parks at T2 (805 m) was significantly greater than the walking distance to parks at T1 (705 m), the difference of 100 m is unlikely to be a deciding factor in park usage.

The strengths of this study include a large sample size and the inclusion of park sizes and walking distances. In addition, the current research was the first to examine similarities/differences between parks that children visit at three different ages. Previous studies have investigated the relationship between age, public open space, and physical activity [24,32], however, children are often grouped into wide ranging age categories such as 6–12 or 3–9 years. This study was unique in that it assessed park use at three distinct timepoints throughout childhood that represent preschool (3–5 years), early primary school (6–9 years) and later primary school years (9–11 years) respectively. Furthermore, this was one of the first studies to assess the relationship between age and features of visited park using a comprehensive audit tool. The tool, purposely developed for this study, allowed for a detailed examination of park features, particularly in relation to features conducive of physical activity (such as playground equipment, sports courts etc.). However, it is important to acknowledge the study limitations. Parents were not asked to report the frequency with which their child visited the park, nor if they also visited other parks. The parks at T1 and T2 were reported retrospectively, and may not accurately reflect the parks that were visited most often at those times and park usage may be culturally specific however this was not considered. Whilst some parks were audited twice for reliability purposes, the vast majority were only audited once, therefore the audit data represent the condition and facilities present at the time of the audit and it is possible that this may have varied over time. It is also possible that certain non-fixed variables, such as the presence of excessive litter or poor maintenance may not have been captured in their typical state. Further, as metropolitan Melbourne is highly developed, and has an abundance of urban parks the findings may not be applicable to children in less developed countries, or those without access to urban parks. Finally, there are a number of limitations in relation to calculating walking distances to parks, and size of parks. Park sizes were obtained using the Victorian Planning Authority’s open access portal. Whilst the portal is one of the most comprehensive and accurate of its kind, Melbourne, like many other cities, is constantly evolving and open spaces are been created, altered and removed as the needs of the population change. Similarly, Google Maps is one of the most well used internet based mapping systems; however, road changes and changes to pedestrian routes mean that there may be some discrepancies between actual walking distance and what was obtained using Google Maps. It is also important to note that data relating to usual travel mode to the park was not collected and this would be a useful inclusion in future studies. Further research may also consider why some children usually visit the park closest to home and others do not, and why some children continue to visit the same park throughout childhood, whilst others do not, particularly in regard to the park features. In addition, future studies could examine whether family characteristics, such as the age of siblings, impact changes in characteristics of parks children visit as they age.

## 5. Conclusions

This research showed that as children reach primary school age, they usually visit parks that are further away from home, and that are larger with more sports and recreation facilities, and other facilities. This research provides practical evidence to inform future park design and re-development and addresses a critical gap in the evidence base as little is currently known about the park characteristics present in parks that children visit and how this changes as children become older. The findings provide timely and novel evidence to assist urban planners, local, state and national governments, as well as park designers, to understand what characteristics of parks are necessary to promote park visitation among children of different ages. Given significant forecasted urban population growth and higher density living, the availability of high quality parks is critical for future generations. Data from this study contributes to knowledge of the optimal design of parks to appeal to children of different ages to ensure park development or refurbishment may have optimal positive effects on park visitation and park-based physical activity to meet the needs of a growing population.

## Figures and Tables

**Table 1:** Descriptive characteristics of the parks at three timepoints.

Park Feature	Data Format	Park Visited at T1 Children: 3–5 Years (*n* = 353)	Park Visited at T2 Children: 6–8 Years (*n* = 375)	Park Visited at T3 Children: 9–11 Years (*n* = 310)
**Access and surrounding neighbourhood**				
Walking distance from home (m)	M *	705.7	805.3	685.6
Park size (ha)	M *	3.9	4.5	4.4
Closest to home	Yes (%)	37.7	34.4	41.0
Public transport within sight	Yes (%)	40.2	44.3	43.5
Road crossing	Yes (%)	37.4	43.2	39.7
Parking	Yes (%)	99.2	99.2	99.7
Bordering roads (>60 Kph)	Yes (%)	7.1	6.7	6.1
Bordering footpaths	Yes (%)	97.7	96.5	95.8
External trail	Yes (%)	35.7	36.8	37.7
**Activity areas within the park**				
Full courts (basketball, netball, tennis etc.)	M (SD)	1.3 (2.9)	1.7 (3.5)	1.7 (3.5)
Half courts (basketball, netball, tennis etc.)	M (SD)	0.4 (0.9)	0.5 (1.0)	0.4 (0.8)
Sports ovals	M (SD)	1.6 (2.8)	2.2 (3.1)	2.2 (3.1)
Other facilities (athletics track, fitness station, swimming pool etc.)	M (SD)	2.4 (2.0)	2.7 (2.0)	2.7 (2.1)
Number of playgrounds	M (SD)	1.2 (0.6)	1.2 (0.6)	1.2 (0.7)
Number of playground equipment	M (SD)	12.9 (6.3)	13.0 (6.5)	12.5 (6.7)
Playgrounds suitable for under 5 years	Yes (%)	94.9	92.2	91.6
Playgrounds suitable for 6–10 years	Yes (%)	92.6	92.3	90.0
Playgrounds suitable for 11–15 years	Yes (%)	26.6	31.5	28.7
Open green space	Yes (%)	99.2	99.7	98.1
**Park quality and safety**				
Toilets	Yes (%)	56.1	61.3	58.4
Drinking fountains	Yes (%)	71.1	74.9	71.3
Benches	Yes (%)	97.2	97.6	96.8
Picnic shelters	Yes (%)	42.2	46.9	42.6
Picnic tables	Yes (%)	85.0	85.1	81.8
Barbeque	Yes (%)	52.4	56.5	51.8
Lights	Yes (%)	65.4	70.7	66.8
Vandalism	Yes (%)	0.8	0.5	1.6
Excessive litter	Yes (%)	7.1	6.9	5.2
Poor maintenance	Yes (%)	1.7	3.2	2.9
Evidence of threatening persons or behaviours	Yes (%)	0.8	1.9	1.0
Signs say dogs must be on a lead	Yes (%)	42.5	37.6	40.3
Signs for dog off lead area	Yes (%)	20.5	24.3	27.2
Signs say dogs must not enter playground	Yes (%)	27.3	25.7	28.2
Evidence of landscaping (e.g., flower beds, formal gardens)	Yes (%)	38.2	39.7	37.7
Artistic features (e.g., statue, fountain)	Yes (%)	24.1	25.1	23.5
Trees	Yes (%)	98.9	99.5	99.0
Water feature (lake, stream, pond etc.)	Yes (%)	28.3	25.9	24.8
Bushland	Yes (%)	21.5	22.7	23.2

Note. M * = geometric mean, M = mean, SD = standard deviation.

**Table 2:** Associations between park features of parks visited compared to previous timepoints.

Park Feature	Statistic	Park Visited at T2 (Ref T1; *n* = 375)	Park Visited at T3 (Ref T1; *n* = 310)	Park Visited at T3 (Ref T2; *n* = 310)
**Access and surrounding neighbourhood**				
Walking distance from home (m)	exp(β) (95% CI)	1.16 (1.03–1.30) *	1.09 (0.96–1.23)	0.94 (0.84–1.06)
Park size (hectares)	exp(β) (95% CI)	1.15 (1.02–1.31) *	1.15 (1.01–1.31) *	0.99 (0.88–1.14)
Closest to home (ref = no)	OR (95% CI)	0.66 (0.40–1.09)	1.10 (0.65–1.85)	1.66 (0.99–2.78)
Public transport within sight (ref = no)	OR (95% CI)	1.54 (0.96–2.48)	1.38 (0.83–2.30)	0.90 (0.55–1.49)
Road crossing (ref = no)	OR (95% CI)	1.89 (1.15–3.10) *	1.31 (0.77–2.22)	0.69 (0.42–1.15)
**Activity areas within park**				
Full courts	IRR (95% CI)	1.42 (1.05–1.92) *	1.32 (0.96–1.81)	0.93 (0.69–1.25)
Half courts	IRR (95% CI)	1.20 (0.96–1.51)	1.17 (0.91–1.50)	0.98 (0.77–1.23)
Sports ovals	IRR (95% CI)	1.36 (1.15–1.60) **	1.33 (1.12–1.59) **	0.98 (0.83–1.16)
Other facilities	IRR (95% CI)	1.10 (1.01–1.21) *	1.09 (0.99–1.20)	n/a
Number of playgrounds	IRR (95% CI)	1.01 (0.88–1.15)	0.99 (0.86–1.14)	0.98 (0.86–1.13)
Number of playground equipment	IRR (95% CI)	1.00 (0.96–1.05)	0.96 (0.92–1.01)	0.96 (0.92–1.01)
Playgrounds suitable for 11–15 years (ref = no)	OR (95% CI)	1.60 (0.98–2.62)	1.20 (0.71–2.04)	0.75 (0.45–1.24)
**Park quality and safety**				
Toilets (ref = no)	OR (95% CI)	1.63 (1.01–2.63) *	1.13 (0.68–1.88)	0.69 (0.42–1.15)
Barbeque (ref = no)	OR (95% CI)	1.56 (0.95–2.55)	0.84 (0.50–1.42)	0.54 (0.32–0.91) *
Lights (ref = no)	OR (95% CI)	1.69 (1.03–2.79) *	1.02 (0.61–1.72)	0.60 (0.36–1.02)
Excessive litter (ref = no)	OR (95% CI)	0.82 (0.20–3.40)	0.08 (0.01–0.53)	n/a
Evidence of threatening persons or behaviours (ref = no)	OR (95% CI)	31.24 (0.32–3089.16)	1.24 (0.07–21.25)	0.04 (0.01–4.33)
Evidence of landscaping (ref = no)	OR (95% CI)	1.17 (0.73–1.86)	1.05 (0.64–1.74)	0.90 (0.55–1.47)
Artistic features (ref = no)	OR (95% CI)	1.20 (0.70–2.07)	1.12 (0.63–2.01)	0.94 (0.53–1.65)
Bushland (ref = no)	OR (95% CI)	1.27 (0.72–2.33)	1.36 (0.74–2.49)	1.07 (0.60–1.92)

*Note*. CI = Confidence intervals, OR = Odds Ratio, IRR = Incidence Rate Ratio, exp(β) = exponentiated coefficient, * *p* < 0.05, ** *p* < 0.01. n/a = could not converge.
